# Non-Thermal Plasma-Enabled Valorization of Sotol Bagasse for Microbial Carotenoid Production

**DOI:** 10.3390/foods15112039

**Published:** 2026-06-05

**Authors:** Itzcoatl Muñoz-Jiménez, Miguel Ángel Villegas-Méndez, Yadira Karina Reyes-Acosta, Alfredo Valentín Reyes-Acosta, Juan Carlos Contreras-Esquivel, Iván Salmerón, Julio Montañez, Lourdes Morales-Oyervides

**Affiliations:** 1 Unidad Sureste, Facultad de Ciencias Químicas, Saltillo 25280, Coahuila, Mexico; itzcoatl_munoz@uadec.edu.mx (I.M.-J.); miguel.villegas@uadec.edu.mx (M.Á.V.-M.); ykreyes@uadec.edu.mx (Y.K.R.-A.); carlos.contreras@uadec.edu.mx (J.C.C.-E.); julio.montanez@uadec.edu.mx (J.M.); 2 Unidad Sureste, Facultad de Sistemas, Saltillo 25000, Coahuila, Mexico; alfredoreyes@uadec.edu.mx; 3 Facultad de Ciencias Químicas, Universidad Autónoma de Chihuahua, Chihuahua 31125, Chihuahua, Mexico; isalmeron@uach.mx

**Keywords:** microbial carotenoids, sotol bagasse valorization, non-thermal plasma, lignin removal

## Abstract

The replacement of synthetic dyes has gained increasing attention due to stricter regulatory policies and growing health concerns. Microbial carotenoids represent a promising alternative to artificial food colorants; however, their large-scale production is limited by the high cost of raw materials. In this context, the valorization of lignocellulosic biomass offers a strategy to develop low-cost substrates for microbial bioprocesses. Sotol bagasse (SB), an underutilized lignocellulosic residue generated during sotol production, was composed of 24% cellulose, 14% hemicellulose, and 42% lignin. A non-thermal plasma pretreatment, optimized through response surface methodology, achieved up to 29% of lignin removal. Subsequent enzymatic hydrolysis yielded a total sugar concentration of 28 g/L. The resulting hydrolysate supported the growth of *Rhodotorula glutinis*, yielding 4.4 g/L of biomass and 0.91 mg/L of carotenoids. To the best of our knowledge, this is the first report describing the use of non-thermal plasma as a pretreatment strategy for sotol bagasse, demonstrating its potential as a chemical-free approach for lignocellulosic valorization and sustainable microbial carotenoid production.

## 1. Introduction

The search for sustainable and health-promoting alternatives to synthetic food colorants has intensified considerably, driven by strict regulations on artificial dyes and increasing consumer awareness of their toxicity [1]. In response, considerable efforts have been directed toward developing natural, cost-effective pigment sources for industrial applications. Carotenoids, in particular, have attracted significant attention due to their antioxidant properties and their wide range of applications across industrial sectors [2]. In this context, microbial production of carotenoids has emerged as a promising approach, offering advantages such as rapid growth, scalability, and independence from environmental constraints [3]. The use of microbial carotenoids in food matrices remains limited; however, their functional properties are comparable to those of carotenoids from conventional sources, enabling their application in food systems such as yogurt, juices, and confectionery products [4]. More recently, the incorporation of microbial carotenoids as natural colorants in gelatin-based products has been reported [5].

Although microbial carotenoids represent a viable alternative, their production costs remain higher than those of synthetic counterparts. For instance, the production cost of synthetic carotenoids is estimated to be approximately 1000 USD per kg, whereas microbial-derived carotenoids can exceed 7000 USD per kg [3]. These higher costs of microbially derived products are primarily due to the composition of the fermentation medium and the relatively low yields from conventional carbon sources, thereby highlighting a significant opportunity to use alternative, renewable feedstocks [6].

Sotol bagasse (SB) is the main by-product generated during the production of the distilled beverage sotol. Approximately 520 m^3^ of sotol are produced annually across the Mexican states of Chihuahua, Coahuila, and Durango, with a projected annual growth rate of 5% [7]. Due to the artisanal nature of the manufacturing process, nearly 9.6 kg of SB are generated per liter of sotol produced [7,8]. SB exhibits a typical lignocellulosic composition and is commonly discarded or incinerated, leading to environmental concerns [9]. Nevertheless, SB has emerged as a suitable feedstock for the production of value-added products, particularly as a carbon source in bioprocess development [10,11]. Despite this potential, its recalcitrant structure necessitates pretreatment strategies to remove lignin and enhance the release of fermentable sugars.

Conventional chemical lignin-removal treatments involve the use of concentrated acids, high pressures, and temperatures, which produce a rich monomeric sugar liquor but also lead to the formation of residual molecules such as acids (acetic, formic, and levulinic), furfural, and hydroxymethyl furfural, which are toxic compounds for microbial growth, inhibiting cellular metabolism and negatively impacting the fermentative processes [12]. On the other hand, novel technologies have attracted attention as alternatives to chemical lignin removal; ultrasound, non-thermal plasma (NTP), and microwaves have been used to treat lignocellulosic biomass, as they offer a feasible approach due to their effectiveness in reducing energy consumption and improving process efficiency [13,14]. Among these, NTP, also known as cold plasma, stands out as a relevant technology due to its ability to modify biomass surfaces, low operational cost, and minimal waste generation, making it an attractive approach to enhance biomass conversion [14]. Other studies have reported the use of NTP for lignin removal from various lignocellulosic biomasses, including brewers’ spent grain [15], sugarcane bagasse [16], hemp fiber [17], and walnut shells [18]. These studies demonstrate the effectiveness of NTP; however, its performance depends on biomass characteristics and treatment configuration. To the best of our knowledge, the application of NTP as a pretreatment strategy for sotol bagasse has not been reported. Therefore, the aim of this study was to evaluate whether non-thermal plasma pretreatment can enable the effective valorization of sotol bagasse by enhancing lignin removal and facilitating its use as a low-cost substrate for microbial carotenoid production.

## 2. Materials and Methods

### 2.1. Raw Material Collection and Characterization

SB was collected from local sotol producers in Chihuahua, Mexico, and oven-dried at 90 °C until the humidity was <5%. The SB was ground (<10 mm particle size) using a rotary mill (Retsch-SM100 Industrial Mill, Haan, Germany). Samples were sieved to eliminate impurities and stored in plastic bags at 25 °C. Proximal analysis of SB was conducted to determine cellulose, hemicellulose, and lignin content.

### 2.2. Non-Thermal Plasma Treatment and Sotol Bagasse Hydrolysis

#### 2.2.1. Plasma Treatment

A low-pressure non-equilibrium cold plasma was generated using a custom-built capacitively coupled plasma reactor powered by a high-frequency resonant transformer. The system operated with a nominal input power of 60 W and an output voltage of 5 kV. Plasma discharge was generated using a sharp electrode in a low-pressure glass reactor (50 mL) filled with residual air, producing a stable, homogeneous plasma treatment environment. For each treatment, 0.1 g of SB was distributed inside the glass reactor. Treatments were carried out in triplicate at an input induction frequency of 500 Hz and an input power of 60 W for 1, 3, and 6 min. Lignin content was determined after plasma treatment to evaluate changes in the lignin fraction of SB.

#### 2.2.2. Optimization of Lignin Removal

To maximize lignin removal from SB via cold plasma treatment, a full factorial experimental design was employed. Frequency and power were defined as independent variables and normalized to coded values to facilitate statistical comparison of their effects, as detailed in Table 1. Power was adjusted as a relative operational setting (%) of the plasma generator rather than as directly measured absorbed power. Treatment duration was fixed at the optimal value identified in Section 2.2.1.

#### 2.2.3. Enzymatic Hydrolysis

An SB hydrolysate was obtained by enzymatic hydrolysis in a 125 mL Erlenmeyer flask containing 1 g of SB (dry mass) in 20 mL of buffer solution (pH 4.8) with 20 FPU of the commercial enzyme blend Cellic^®^ CTec2. Samples were incubated at 50 °C for 24 h in an orbital shaker (New Brunswick Scientific Co., Inc., Edison, NJ, USA) at a constant stirring speed of 200 rpm. The total sugar concentration of the hydrolysate was determined after the incubation.

### 2.3. Inoculum Preparation and Carotenoid Production

The yeast *Rhodotorula glutinis* PM422, provided by the Chemical Engineering Department of Universidad Autonoma de Coahuila, was employed for carotenoid production. The strain was cultivated in Petri dishes using YM medium (g/L): glucose, 10; peptone, 5; yeast extract, 3; malt extract, 3; and agar, 15, at 30 °C for 48 h. Subsequently, a single colony was transferred to 125 mL Erlenmeyer flasks containing 25 mL of YM broth (g/L): glucose, 10; peptone, 5; malt extract, 3; and yeast extract, 3. Inoculum broth was obtained after incubating the flasks at 30 °C with constant agitation at 180 rpm for 24 h. Carotenoid production was carried out in 125 mL Erlenmeyer flasks using SB hydrolysate adjusted to 16.71 ± 1.33 g/L of initial total sugars and supplemented with the following components (g/L): yeast extract, 2; KH_2_PO_4_, 7; Na_2_HPO_4_, 2.5; MgSO_4_, 1.5; CaCl_2_•2 H_2_O, 0.15; FeCl_3_∙6 H_2_O, 0.15; ZnSO_4_∙7 H_2_O, 0.02; and MnSO_4_∙H_2_O, 0.06. The medium pH was adjusted to 5.0 before autoclaving. Once cooled, the medium was inoculated with a 10% (*v*/*v*) broth culture. The yeast fermentation was carried out in an orbital shaker at 200 rpm, 30 °C for 96 h.

### 2.4. Analytical Methods

#### 2.4.1. Compositional Analysis of Sotol Bagasse

The hemicellulose, cellulose, and lignin contents of SB were determined according to the NREL/TP-510-42618 procedure [19]. Briefly, the determination of cellulose and hemicellulose fractions was as follows: SB was hydrolyzed with 72% sulfuric acid at 30 °C for 1 h, then the acid was diluted (3% *v*/*v*) and autoclaved at 121 °C (1 atm) for 1 h. Finally, samples were vacuum-filtered using a porous glass filter. The liquid fraction was analyzed by high-performance liquid chromatography on an Agilent Technology 1200 series chromatograph (Agilent, Santa Clara, CA, USA) equipped with a Bio-Rad HPX87H (300 mm × 7.8 mm I.D.) column and a refractive index detector at 45 °C. The mobile phase was 5 mM H_2_SO_4_ at a flow rate of 0.6 mL/min. The concentrations of glucose, xylose, and arabinose were quantified and subsequently used to calculate the cellulose and hemicellulose content of the biomass. The lignin content was determined as the sum of acid-insoluble and acid-soluble fractions. The solid fraction remaining after acid hydrolysis was dried at 105 °C to constant weight and quantified as acid-insoluble lignin. For the determination of acid-soluble lignin, the liquid fraction was diluted 1:10 (*v*/*v*) with deionized water, and its absorbance was measured at 205 nm using a UV/Vis spectrophotometer (UNICO 2150, Dayton, NJ, USA).

#### 2.4.2. Total Sugar Determination

TS concentration was measured using the phenol sulfuric acid method [20]. Previously collected supernatants from hydrolysis and fermentation were mixed with 5% (*w*/*v*) phenol and concentrated sulfuric acid. The mixtures were incubated in boiling water (100 °C) for 10 min, then cooled for 10 min in an ice bath; afterward, the absorbance was measured with a spectrophotometer at 490 nm.

#### 2.4.3. Determination of Yeast Dry Cell Weight

Yeast fermentation broth was collected into a centrifuge tube for biomass analysis. The tubes were centrifuged (10,000 rpm at 4 °C) for 10 min, and the biomass was washed twice with distilled water. Biomass concentration was quantified as dry cell weight after oven-drying the samples at 80 °C until constant weight.

#### 2.4.4. Carotenoids Quantification

Total carotenoids were quantified following the methodology described by Villegas-Mendez et al. (2023) [21]. For this purpose, yeast biomass was disrupted by freezing, followed by the addition of preheated dimethyl sulfoxide to 55 °C and subsequent homogenization of the cellular suspension. The carotenoid fraction was extracted by adding petroleum ether, acetone, and NaCl solution (20% *w*/*v*) to the disrupted cells. After phase separation, the petroleum ether fraction was collected, and its absorbance was measured at 450 nm. Total carotenoid was calculated using Equation (1) and expressed as β-carotene equivalents [21,22,23]:

(1) $$Yx=\frac{V\times A\times 10^{4}}{E^{1\%}\times ms}$$

where *Y_x_*, carotenoids accumulation (μg/g, dry weight); *A*, absorbance; *V*, volume of solvent used in mL; ms, dry cell mass (g); *E*^1%^, specific absorptivity of β-carotene in petroleum ether (2592).

### 2.5. Data Analysis

Statistical analysis was performed using analysis of variance (ANOVA) and the least significant difference post hoc test for multiple comparisons. Results were considered significantly different at a 95% confidence level (*p* < 0.05).

The experimental data from the lignin removal optimization stage were fitted to a second-order polynomial model:

(2) $$R=\beta_{o}+\beta_{1}F_{1}+\beta_{2}F_{2}+\beta_{11}{F_{1}}^{2}+\beta_{22}{F_{2}}^{2}+\beta_{12}F_{1}F_{2}$$

where *R* represents the response variable (Lignin removal, %); $F_{1}$ and $F_{2}$ are the coded levels of frequency and power, respectively. The coefficients are for the intercept ($\beta_{o}$), linear ($\beta_{1}$, $\beta_{2}$), quadratic ($\beta_{11}$, $\beta_{22})$ and interactive effects $\left(\beta_{12}\right)$ of each factor.

Model fitting was performed using Microsoft Excel (Microsoft Excel version 2010, Microsoft Corporation), while statistical analyses were conducted using Statistica 8.0 (StatSoft, Tulsa, OK, USA).

## 3. Results

### 3.1. Sotol Bagasse Characterization

Table 2 compares the structural composition of characterized SB obtained in this study with values reported in the literature. The cellulose content was lower than previously reported, whereas hemicellulose values fell within the expected range. In contrast, SB exhibited a notably higher lignin content compared to other studies.

These differences highlight the intrinsic variability of SB as a lignocellulosic feedstock, which may be influenced by factors such as plant maturity, geographic origin, and processing conditions during sotol production. Given that sotol is primarily produced through artisanal methods, variations in operational practices may further contribute to compositional heterogeneity [9]. Similar variability has been reported for other agro-industrial lignocellulosic residues, reinforcing the importance of detailed compositional characterization prior to their integration into biorefinery processes [12].

Based on its composition, the SB analyzed in this study can be classified as a high-lignin lignocellulosic biomass (>30% lignin content). This type of biomass is typically associated with increased structural recalcitrance, which limits enzymatic accessibility and requires more intensive pretreatment strategies [24].

High-lignin feedstocks are often considered more suitable for thermochemical conversion routes, such as pyrolysis or gasification, whereas their integration into biochemical processes is more challenging due to the need for effective delignification [25]. However, if SB is to be incorporated into a bioprocess aimed at generating value-added compounds, aggressive pretreatments may not be desirable, as they can increase process complexity and promote the formation of compounds that negatively affect downstream microbial conversion.

In this context, the use of non-thermal plasma under relatively mild conditions represents an attractive alternative for SB valorization, as it enables structural modification of the biomass while remaining compatible with subsequent biochemical conversion.

### 3.2. Non-Thermal Plasma Pre-Treatment

Non-thermal plasma (NTP) treatment was evaluated as a pretreatment strategy for SB delignification and to enhance its susceptibility to enzymatic hydrolysis. Although plasma treatment may also promote lignin oxidation and structural modification in lignocellulosic biomass [14,17,18], the reductions reported in this study correspond to decreases in the quantified lignin fraction determined by compositional analysis. The results of the preliminary experiments (Table 3) showed that lignin removal reached up to ≈32%. Nevertheless, the differences among the evaluated exposure times were minimal, suggesting that extending the plasma treatment did not substantially improve lignin removal.

These results suggest that most of the measurable reduction in lignin content occurs during the initial plasma exposure, and that extending the treatment time does not substantially improve delignification. This indicates that NTP may rapidly induce structural changes in biomass, rendering prolonged exposure unnecessary.

This behavior is consistent with previous reports showing that non-thermal plasma can induce rapid, surface-driven lignin depolymerization within the first minutes of treatment. This effect has been attributed to the preferential cleavage of labile lignin linkages, such as β-O-4 bonds, as well as to the action of reactive oxygen species generated during plasma discharge [26]. Furthermore, the limited improvement observed at longer treatment times may be explained by the occurrence of competing reactions, including oxidation and potential re-condensation of lignin fragments, which can reduce the net delignification effect despite continued plasma exposure.

Similar trends have been reported in other lignocellulosic substrates. For example, in brewers’ spent grain, NTP treatment achieved 42% delignification within the first 2.5 min, with only a marginal increase at longer treatment times [27]. Although higher delignification levels have been reported under different plasma configurations or longer treatment durations, such as 36.03% removal after 10 min [15] or 58.5% lignin solubilization after 2 h in sugarcane bagasse [16]. These differences are likely associated with variations in biomass compositions, treatment conditions, and plasma configuration.

Additionally, the NTP system employed in this study was designed at laboratory scale, where the low SB loading (0.1 g) facilitated a more homogeneous plasma–biomass interaction during treatment. In this context, the short exposure times observed may represent an operational advantage by minimizing pretreatment residence time requirements. Nevertheless, scaling this approach toward higher biomass capacities will require further evaluation of plasma distribution, treatment uniformity, and energetic efficiency under larger-volume or continuous reactor configurations [16].

Mechanistically, the literature suggests that plasma treatment may modify lignin through a combination of physical and chemical effects. Energetic particles generated during the discharge can disrupt plant cell structures by breaking the hydrogen bonds within the lignocellulosic matrix [28]. In addition, plasma-generated reactive oxygen species, such as hydroxyl radicals and ozone, have been reported to degrade aromatic rings and double bonds in lignin, thereby facilitating its depolymerization [27].

**Figure 1:**
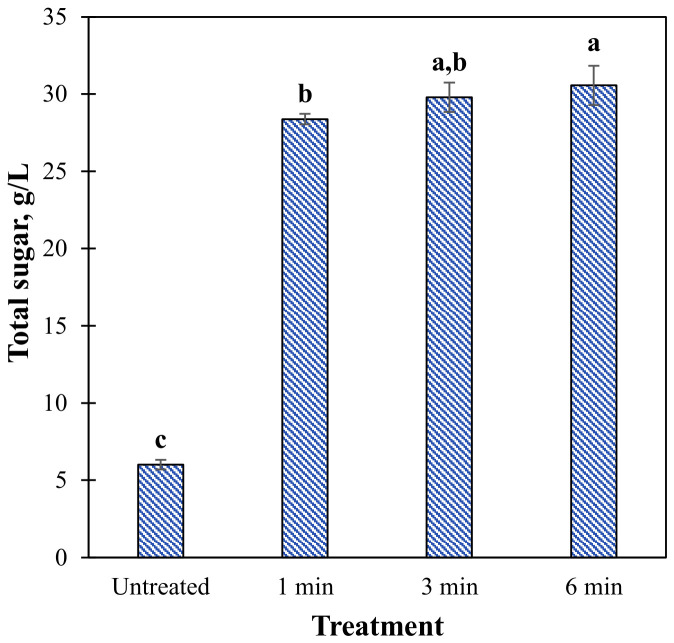
Total sugar release (g/L) after enzymatic hydrolysis of non-treated and NTP-treated samples. Lowercase letters (a–c) indicate statistically difference among treatments (*p* < 0.05).

Plasma pretreatment influenced the efficiency of enzymatic hydrolysis of SB. As shown in Figure 1, treated samples exhibited increased sugar release compared with untreated biomass. After 24 h of enzymatic hydrolysis, raw SB released 6.00 ± 0.31 g/L, whereas plasma-treated samples released up to 30.56 ± 1.28 g/L. This represents a >5-fold increase in sugar availability. Similar trends have been reported by Ravindran et al. (2017), who observed a 1.7-fold increase in reducing sugars from plasma-treated coffee spent waste [29] as well as a 2.14-fold increase in sugar release from plasma-treated brewers’ spent grain [15]. In addition to improving sugar release, plasma pretreatment combined with enzymatic hydrolysis has been associated with reduced formation of inhibitory degradation products compared with conventional chemical-based pretreatments [14,15,16]. Although detailed hydrolysate characterization was not performed in the present study, the enhanced enzymatic hydrolysis and increased sugar release observed after plasma treatment suggest that the resulting hydrolysate could be suitable for subsequent bioconversion processes. Nevertheless, comprehensive analysis of individual sugars and potential inhibitory compounds will be necessary to further assess hydrolysate suitability for fermentation applications.

In addition to reducing the quantified lignin fraction, plasma treatment can modify the surface properties of lignocellulosic materials by increasing surface area and porosity, thereby enhancing enzyme accessibility [17]. Furthermore, the increased sugar release observed at longer treatment durations (Figure 1), despite similar lignin removal levels, suggests that plasma exposure may also promote additional structural modifications within the biomass matrix, possibly including changes in cellulose crystallinity. Anari et al. (2024) reported that plasma treatment promoted the disruption of cellulose crystalline regions of walnut shells [18]. Although similar effects may have occurred in the present study, further analytical characterization would be necessary to confirm these modifications. Also, NTP has been reported to increase the hydrophilicity of fibrous materials, facilitating the diffusion of enzyme solutions into the substrate and promoting more effective biocatalytic interactions [28].

Based on these results, a treatment time of 3 min was selected for subsequent experiments because it provided enhanced sugar release while maintaining treatment duration within a moderate operational range. Since the objective of this study was to evaluate the feasibility of SB valorization through plasma-assisted pretreatment and microbial conversion, this condition was considered suitable for further exploring the effects of frequency and power on biomass modification and hydrolysis performance.

### 3.3. Optimization of Lignin Removal by Plasma Treatment

The optimization of lignin removal was evaluated by analyzing the effect of NTP operating conditions on SB delignification efficiency. The results obtained under the evaluated conditions are presented in Table 4, while the corresponding ANOVA results are summarized in Table 5. The maximum lignin removal (28.73 ± 0.07%) was achieved in run 4, using 750 Hz and 60%. In contrast, the lowest removal (24.85 ± 1.92%) was observed at 1000 Hz and 60%.

**Figure 2:**
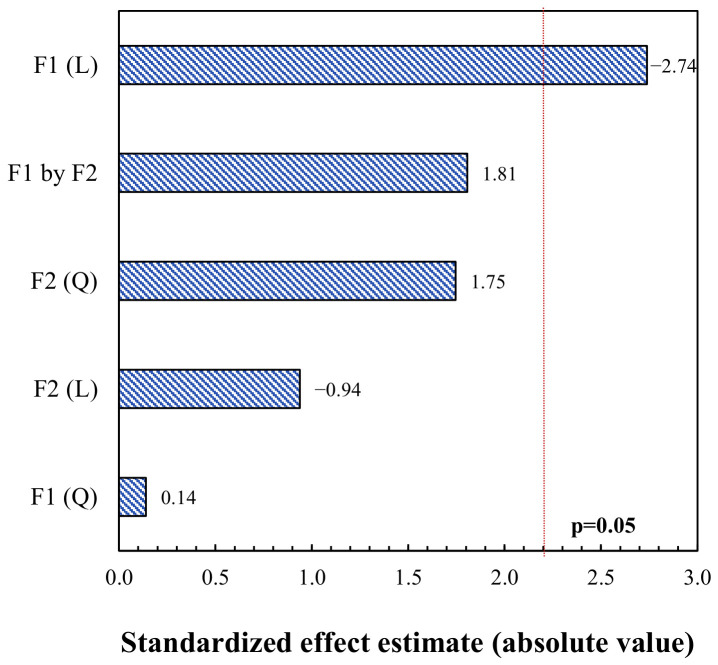
Pareto chart of frequency, power, and their interaction effects on the lignin removal. The red dashed line indicates statistical significance (*p* < 0.05).

As shown in Figure 2, the Pareto chart indicates that frequency (linear effect) was the only statistically significant factor (*p* < 0.05) among the evaluated variables. Notably, frequency had a negative effect on lignin removal, indicating that increasing frequency led to a decrease in delignification efficiency. In contrast, power did not show a significant individual effect within the studied range. This negligible effect of power may be attributed to the high lignin content of SB, which could deplete the reactive species generated during plasma treatment [14]. Previous studies have demonstrated that the disruption of lignin–cellulose interactions is governed by the applied discharge characteristics, such as operating voltage, rather than by increases in power input [18,28].

Although the interaction between frequency and power was not statistically significant, its standardized effect exceeded that of the relative power setting alone.

In any case, a second-order polynomial model was fitted to describe the relationship between the evaluated variables and lignin removal. The model can be expressed as

Lignin removal (%) = 25.86 − 1.30F_1_ − 0.42F_2_ + 0.11F_1_^2^ + 1.40F_2_^2^ + 1.02F_1_F_2_ (3)

The model showed a satisfactory fit, with a coefficient of determination (R^2^) of 0.88, indicating that it adequately describes the experimental data within the evaluated range.

**Figure 3:**
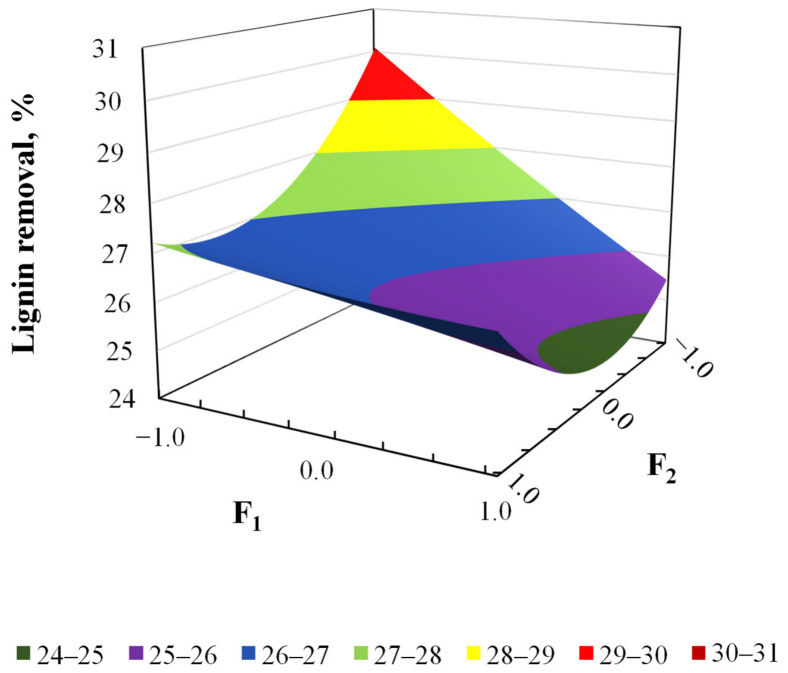
Response surface plot for lignin removal using plasma treatment (F_1_, frequency; F_2_, power).

Although only the linear effect of F_1_ was statistically significant, the response surface was generated using the full model to visualize the combined effect of frequency and power on lignin removal (Figure 3). As observed, lignin removal decreases with increasing frequency, confirming its linear effect. Variations associated with the relative power setting appeared comparatively limited within the evaluated range, although slight changes in the response surface profile were observed at lower power conditions. Therefore, interpretations of possible interactions between factors should be treated as qualitative trends rather than statistically validated effects.

According to the model, the optimal region for lignin removal was identified at F_1_ = −1 and F_2_ = −1, corresponding to a frequency of 500 Hz and operational power of 60%. Under such conditions, the predicted lignin removal was 29.36 2.13%.

Interestingly, the optimal conditions identified by the model maintained the same frequency used in the preliminary experiments while requiring a lower operational power setting, suggesting that increasing process severity does not necessarily lead to improved delignification under the evaluated conditions.

Beyond identifying optimal conditions, the model provides insight into the roles of the evaluated variables in the plasma–SB interaction. The negative effect of frequency indicates that increasing frequency reduces delignification efficiency, suggesting that discharge characteristics play a key role in the effectiveness of energy transfer to the biomass. In contrast, variations in the relative power setting produced limited effects on lignin reduction within the evaluated operational range. Nevertheless, considering the relatively high lignin content of SB (>40%) compared with other reported lignocellulosic substrates [15,28], the lignin reduction achieved in this study supports the effectiveness of the evaluated NTP conditions for the pretreatment of highly lignified biomass.

In general, plasma power and frequency are recognized as key parameters controlling lignin removal efficiency, as they influence electron energy, discharge intensity, and the generation of reactive species responsible for lignin depolymerization [30,31].

Higher power and frequency are typically associated with an increased density of reactive species and enhanced cleavage of lignin bonds. However, both parameters have been reported to exhibit optimal operating ranges, as excessively high power or frequency can promote over-oxidation or competing side reactions that ultimately limit effective lignin removal [30,32,33]. In this sense, the behavior observed in this study aligns with the notion that delignification efficiency is not solely enhanced by increasing discharge intensity but rather depends on maintaining suitable plasma conditions that favor selective lignin modification. Therefore, the impact of frequency should be considered not only as a means of increasing energy input but also in how it influences the plasma–biomass interaction dynamics.

These observations indicate that lignin modification under the evaluated conditions is not limited by the amount of energy supplied, but rather by how that energy is delivered through the plasma. Therefore, frequency appears to be the main parameter controlling the effectiveness of plasma-induced structural changes in the biomass.

**Figure 4:**
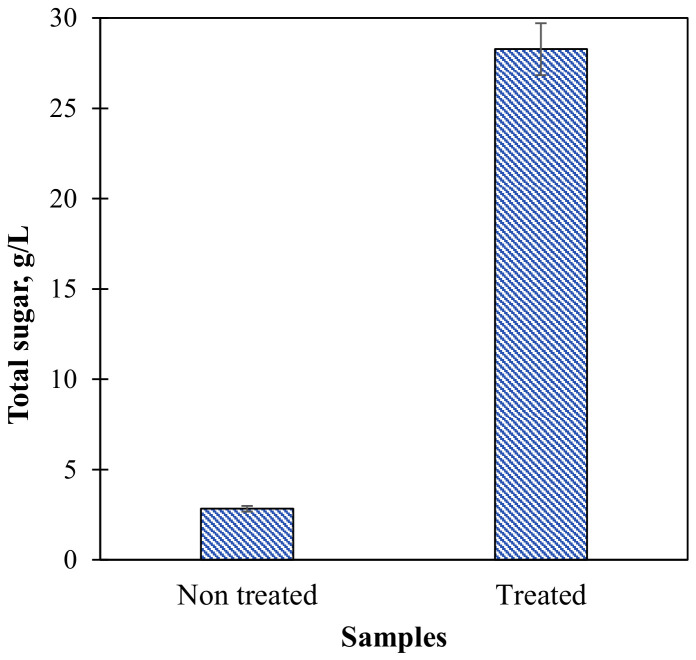
Comparison of total sugar release from untreated and plasma-treated sotol bagasse after enzymatic hydrolysis.

To validate the optimized plasma conditions predicted by the RSM model, enzymatic hydrolysis was performed using plasma-treated and untreated sotol bagasse. As shown in Figure 4, plasma pretreatment under the selected conditions resulted in a total sugar concentration of up to 28.27 ± 1.44 g/L, which was significantly higher than that obtained from untreated biomass. Notably, the sugar release obtained under the optimized conditions was comparable to that observed during the preliminary assays despite the lower relative power setting employed during optimization. This behavior implies enhanced SB digestibility, thereby increasing total sugar availability after plasma treatment. Similar trends have been reported for other lignocellulosic residues. For example, Miranda et al. (2020) reported approximately 17 g/L of total sugars from plasma-treated sugarcane bagasse after enzymatic hydrolysis [16]. Similarly, a hydrolysate obtained from plasma-treated brewer’s spent grain yielded 162.9 mg of reducing sugars per gram of biomass [15].

The higher sugar yield observed in this study (0.57 g/g of SB) can be attributed to the structural modifications induced by plasma treatment, which likely enhanced enzyme accessibility to carbohydrate fractions. These effects may include partial lignin disruption, increased porosity, and alterations in the lignocellulosic matrix, rather than lignin removal alone. Furthermore, the sugar concentration obtained (28 g/L) falls within a range suitable for microbial processes, as oleaginous yeasts such as *Rhodotorula glutinis* have been reported to grow efficiently at initial sugar concentrations as low as ~10 g/L [34]. This highlights the potential of the obtained hydrolysate as a feasible substrate for subsequent bioprocess design.

### 3.4. Carotenoid Production Using Sotol Bagasse Hydrolysis

The sugars obtained from enzymatic hydrolysis were successfully used as a carbon source to support both growth and carotenoid production by an oleaginous yeast. The microorganism consumed 75% of the initial substrate concentration, corresponding to 12.57 ± 1.33 g/L.

This sugar consumption resulted in a maximum biomass concentration of 4.4 ± 0.4 g/L, corresponding to a biomass yield on substrate of 0.35 ± 0.04 g/g. Additionally, a total carotenoid concentration of 0.91 ± 0.07 mg/L was obtained, with a carotenoid content of 0.21 ± 0.02 mg/g biomass. Overall, the microorganism produced 0.074 ± 0.008 mg of carotenoids per gram of sugar consumed. Although the specific carotenoid profile was not characterized by HPLC, previous studies have shown that *Rhodotorula* species cultivated on lignocellulosic hydrolysates typically produce a carotenoid profile composed mainly of β-carotene, torulene, and torularhodin [35,36]. The production of these carotenoids further supports the potential of lignocellulosic hydrolysates as substrates for generating value-added bioactive compounds [4,37].

Given the scarcity of literature on carotenoid production from SB hydrolysate, our results were compared with studies on other lignocellulosic feedstocks. Table 6 summarizes biomass and carotenoid yield parameters for various wild-type yeast carotenoid-producing strains.

Notably, the carotenoid yield obtained in the present study falls within the range reported for wild-type strains such as *Xanthophyllomyces dendrorhous* and *Rhodotorula babjevae* using sugarcane bagasse hydrolysate [38,39]. In contrast, higher carotenoid accumulation has been reported for *Rhodosporidium glutinis*, reaching up to 20 mg/g of biomass when cultivated on sugarcane hydrolysate [40]. Such differences may be attributed to variations in hydrolysate composition and to differences in culture optimization strategies, including nitrogen supplementation of medium components, such as the nitrogen source [39,40]. Nevertheless, optimized carotenoid production systems typically rely on defined media and efficient cultivation conditions [2,4], whereas the use of a complex and highly recalcitrant agro-industrial residue such as SB may impose additional metabolic constraints on microbial growth and pigment biosynthesis. Although the carotenoid concentrations obtained in the present study remain lower than those reported for industrially optimized microbial carotenoid production systems, the results demonstrate the feasibility of integrating plasma-assisted pretreatment, enzymatic hydrolysis, and microbial fermentation for the valorization of sotol bagasse. It is important to note that the present work was conceived as a proof-of-concept study rather than a fully optimized fermentation process. Therefore, additional improvements in hydrolysate composition, nutrient supplementation, cultivation strategy, and microbial performance could further enhance carotenoid productivity and support future development of this integrated biorefinery approach.

**Table 6:** Comparison of biomass production and carotenoid yields of yeast strains grown on lignocellulosic hydrolysates.

Strain	Hydrolysate Source	Biomass, g/L	Carotenoid Content, mg/g	Reference
*Rhodotorula glutinis* PM 422	Sotol bagasse	4.4 ± 0.4	0.21 ± 0.02	This work
*Rhodosporidium* *toruloides* NRRL Y-1091	Wheat straw	17.95 ± 0.42	1.37 ± 0.07	[41]
*Rhodosporidium* *toruloides* ACCC 20341	Tea waste	11.85 ± 0.49	16.83 ± 0.08	[42]
*Xanthophyllomyces dendrorhous* ATCC 24202	Sugarcane bagasse	-	0.46	[38]
*Rhodotorula babjevae* IBRC-M30088	Sugarcane bagasse	9.6 ± 0.20	0.14 ± 0.11	[39]
*Rhodotorula glutinis* CCT-2186	Sugarcane bagasse	5.8 ± 0.06	20.43	[40]

Therefore, although this study represents a first approach to the use of plasma-treated sotol bagasse hydrolysate for carotenoid production, the results obtained are promising. Further optimization of culture conditions, medium composition, and process parameters could enhance metabolite production and support the development of a more efficient and robust bioprocess.

**Figure 5:**
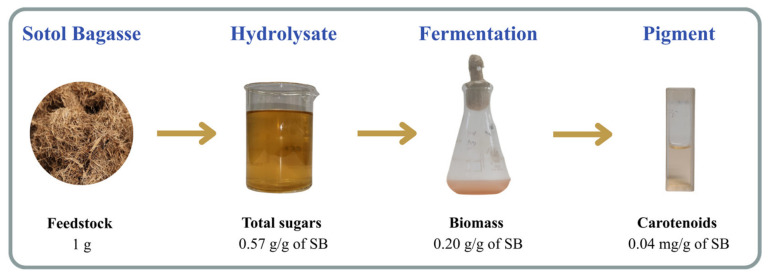
Material balance for the conversion of sotol bagasse into sugars, biomass, and carotenoids, expressed per 1 g of initial feedstock.

To further support the potential of SB hydrolysate and based on the hydrolysis and fermentation yields obtained in this study, a simplified mass balance was established using 1 g of sotol bagasse as the calculation basis. According to the integrated process, 1 g of SB can yield 0.57 g of total sugars, which can be further converted into 0.2 g of yeast biomass and 0.04 mg of carotenoids (Figure 5).

Although the overall yield on a substrate basis appears low, direct comparison with literature remains limited, as carotenoid production is rarely reported per gram of lignocellulosic residue and is typically expressed in terms of microbial biomass or volumetric yields. For instance, Liu et al. (2020) reported that 1 g of wheat straw yields approximately 1 mg of carotenoids [41]. This lack of standardized reporting prevents meaningful comparison at the process level. In this context, the material balance provides a more realistic assessment of overall performance and highlights key opportunities for process improvement toward scale-up.

## 4. Conclusions

Sotol bagasse represents an underutilized raw material with significant potential for conversion into value-added products. The integration of non-thermal plasma pretreatment, enzymatic hydrolysis, and microbial fermentation enabled enhanced sugar release and subsequent carotenoid biosynthesis using an oleaginous yeast. In this context, plasma-assisted pretreatment under mild operational conditions emerges as a promising alternative for biomass modification while reducing reliance on severe chemical treatments.

The hydrolysate generated from plasma-treated SB supported microbial growth and carotenoid production despite the inherent recalcitrance commonly associated with lignocellulosic substrates. However, the present work was conducted under laboratory-scale conditions, and additional efforts involving reactor configuration, hydrolysate characterization, and cultivation optimization will be necessary to further evaluate process performance under increased biomass loading and larger treatment scales.

Overall, the proposed approach contributes to ongoing efforts aimed at developing alternative strategies for lignocellulosic biomass valorization through plasma-assisted pretreatment and microbial bioconversion.

## Figures and Tables

**Table 1:** Independent variables and experimental levels for the plasma-assisted delignification process.

Factor	Level		
	−1	0	1
F_1_ Frequency, Hz	500	750	1000
F_2_ Power, %	60	80	100

**Table 2:** Compositional analysis of sotol bagasse compared with the literature.

Cellulose (%)	Hemicellulose (%)	Lignin (%)	Reference
24.30 ± 0.90	14.10 ± 0.20	42.00 ± 3.60	This study
31.14	3.11	35.04	[10]
38.11 ± 1.48	24.21 ± 0.97	18.78 ± 0.82	[9]
37.74 ± 1.86	25.66 ± 1.86	29.08 ± 2.04	[11]

**Table 3:** Effect of non-thermal plasma treatment time on lignin removal.

Treatment Time, Min	Lignin Removal (%)
1	32.6 ± 3.1
3	32.7 ± 1.3
6	32.1 ± 0.4

**Table 4:** Experimental design matrix and lignin removal responses.

Run	Factor Level		Lignin Removal, %
	F_1_	F_2_	
1	−1.00	−1.00	26.95 ± 3.17
2	−1.00	0.00	27.15 ± 1.85
3	−1.00	1.00	27.77 ± 0.54
4	0.00	−1.00	28.73 ± 0.07
5	0.00	0.00	25.73 ± 0.89
6	0.00	1.00	25.89 ± 1.32
7	1.00	−1.00	24.85 ± 1.92
8	1.00	0.00	25.11 ± 0.00
9	1.00	1.00	27.05 ± 2.07

**Table 5:** Analysis of variance of the response surface model for non-thermal plasma treatment in sotol bagasse.

Variable	SS	DF	MS	F	*p*
F_1_ (L)	15.77	1	15.77	7.50	0.02
F_1_ (Q)	0.04	1	0.042	0.02	0.89
F_2_ (L)	1.85	1	1.85	0.88	0.37
F_2_ (Q)	6.40	1	6.41	3.05	0.11
F_1_ by F_2_	6.86	1	6.86	3.26	0.10
Error	21.01	10	2.10		
Total SS	44.08	15			

L, linear; Q, quadratic.

## Data Availability

The original contributions presented in the study are included in the article, further inquiries can be directed to the corresponding author.

## Abbreviations

The following abbreviations are used in this manuscript:

NTP	Non-Thermal Plasma
SB	Sotol Bagasse
